# Pleomorphic Adenomas of the Parapharyngeal Space

**DOI:** 10.1155/2014/168401

**Published:** 2014-07-22

**Authors:** İstemihan Akın, Tuğba Karagöz, Murad Mutlu, Mehmet Şahan, Evrim Önder

**Affiliations:** ^1^Department of Otorhinolaryngology and Head and Neck Surgery, Faculty of Medicine, Kafkas University, Kars, Turkey; ^2^Department of Otorhinolaryngology and Head and Neck Surgery, Diskapi Yildirim Beyazit Training and Research Hospital, Ministry of Health, Ankara, Turkey; ^3^Department of Pathology, Diskapi Yildirim Beyazit Training and Research Hospital, Ministry of Health, Ankara, Turkey

## Abstract

*Background.* Parapharyngeal space is one of potential facial planes for neoplasms and infections and represents less than 1% of all head and neck tumours. Occurrence of the pleomorphic adenoma in the parapharyngeal space is a rarity. *Case Presentation.* Here, three giant pleomorphic adenomas of different sizes occupying the parapharyngeal space in three patients are reported. Extensive preoperative diagnostic workup was done in order to verify the nature and size of the tumour and the proximity to the large vessels. Review of the literature, clinical features, pathology, radiological findings, and treatment of these tumours are discussed. *Conclusion.* The excision of the tumor through submandibular transcervical approach, without cutting the mandible, turned out to be a safe and radical approach in all three cases.

## 1. Introduction

Parapharyngeal space (PPS) resembles an inverted triangular pyramid with concave faces. This space is located posterior to infratemporal fossa anteriorly, nasopharynx and the lateral pharyngeal wall medially, vertebral column posteriorly, and mandibular ramus laterally. The base of the pyramid is situated on the skull base and the apex is found where the posterior digastric muscles and the greater cornu of hyoid bone meet. The space is further divided into prestyloid and poststyloid compartments by styloid process and its attached muscles and fascia. Posterior to the styloid musculature lies the carotid artery with jugular vein and sympathetic chain with cranial nerves IX through XII.

PPS is one of potential facial planes of head and neck that may become involved by various pathological processes: infectious, inflammatory, and neoplastic. Neoplastic tumours seen at the PPS represent less than 1% of all head and neck tumours. Both benign and malignant tumors may arise from any structure contained within the PPS where 70–80% appears to be benign and 20–30% appears to be malignant. Most of the tumours arising from the posterior compartment are of neurogenic origin while salivary gland tumours are predisposing the anterior compartment.

Most PPS tumors are of salivary gland tumors, neurogenic tumors especially Schwannomas and paragangliomas, and lymphoreticular lesions which comprise nearly 80% of PPS tumors. The most common tumors arising in the PPS are of salivary gland origin, which account for 40–50% of PPS lesions and are located in the prestyloid PPS. These tumors may originate either in deep lobe of parotid gland, in ectopic salivary gland nests, or in minor salivary glands of the lateral pharyngeal wall. The most common prestyloid PPS lesion is “pleomorphic adenoma,” which represents 80–90% of salivary neoplasms in the PPS.

In this paper three cases of PPS pleomorphic adenomas and their treatment are reported.

## 2. Case 1

A 50-year-old male presented with dysphagia and progressive painless swelling in the left submandibular region of 4-month duration. As the mass mimicking peritonsillar abscess, it had been drained in the previous Ear Nose Throat clinic and was given narrow antibiotics for a week.

**Figure 1:**
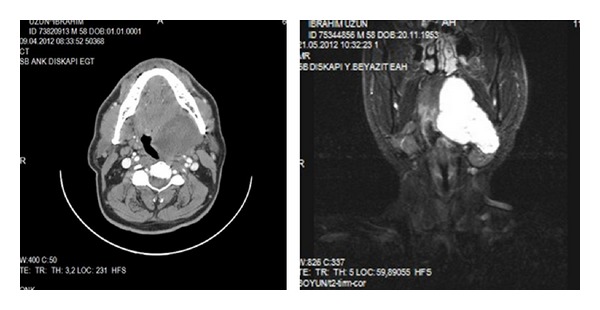
Preoperative CT scan and MRI demonstrate a hyperintense mass.

**Figure 2:**
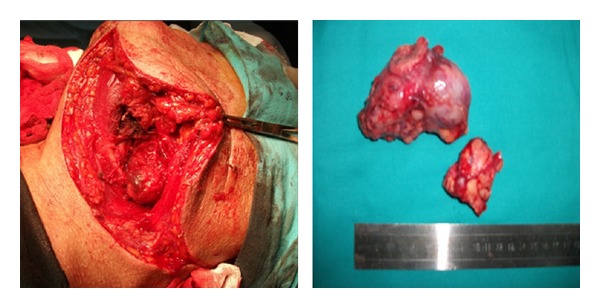
Intraoperative and gross feature of the mass.

**Figure 3:**
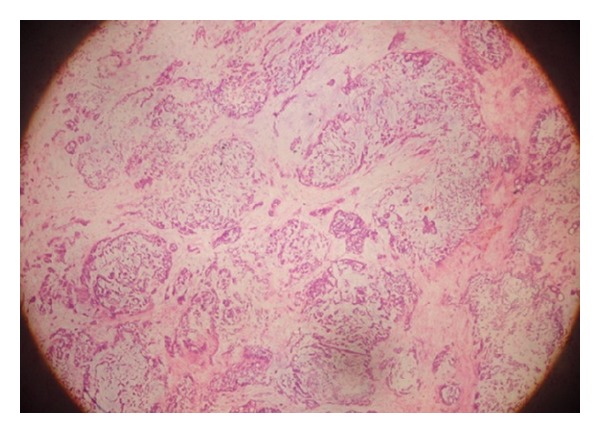
The histological features of the pleomorphic adenoma (40 H&E).

On intraoral examination of the patient, there was a firm bulging of the left soft palate and left lateral pharyngeal wall crossing the midline and pushing the uvula to right side along with a smooth overlying mucosa. Digital palpation of the left tonsil was felt to be firm as well. Although the nontender lesion of the patient caused a considerable degree of dysphagia, no major complaints were reported. He had no fever and denied soreness of his throat. The mass pushing the left tonsil towards the midline of the oropharynx was extended from the left side of nasopharynx to the oropharynx. Externally, there was a swelling in the left submandibular region. Clinical examination did not reveal involvement of any of the cranial nerves. With a clinical diagnosis of parapharyngeal space tumour CT and MRI scan were taken which showed homogenously enhancing tumour measuring 5 × 4 cm in the left parapharyngeal space (Figure 1). Fine needle aspiration cytology of the mass in the neck was consistent with benign mixed tumour. A transcervical approach was used to gain access to the left parapharyngeal space without any mandible osteotomy. The tumour was completely excised. On gross examination the lesion was 9 × 6 × 2.5 cm (Figure 2). Histopathological examination showed a neoplasm having an admixture of epithelial and stromal components (Figure 3).

## 3. Case 2

**Figure 4:**
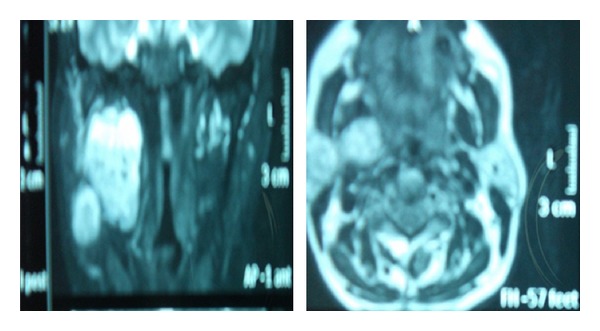
MR image shows the involving of the deep lobe of the right parotid gland.

A 47-year-old female was referred to our clinic with a complaint of swelling being around 3 × 4 cm in diameter, of solid consistency, nonmobile, and not painful when palpated in the lower pole of the right parotid gland. Confirmed by FNAC showing admixed epithelial, myoepithelial, and mesenchymal components consistent with pleomorphic adenoma, the lesion was found to be well defined and encapsulated at magnetic resonance imaging (Figures 4(a) and 4(b)). The lesion was surgically removed by total parotidectomy via a classic transparotid nerve-sparing technique. No complications were observed four years of follow-up after surgery.

## 4. Case 3

**Figure 5:**
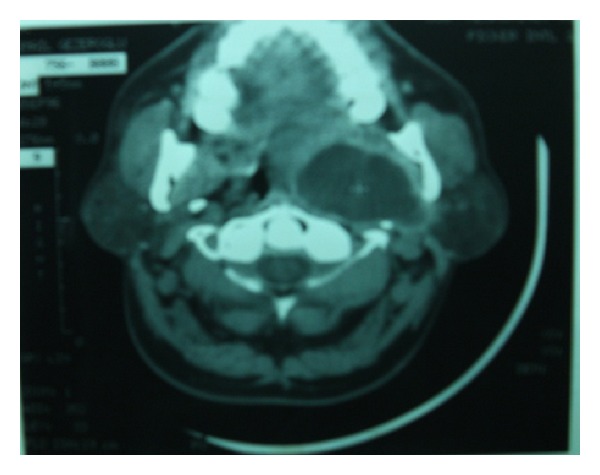
Axial CT scan.

A 56-year-old male presented with fullness in the left side of his throat for a year. Intraoral examination showed occupying the left soft palate and left lateral pharyngeal wall crossing the midline and pushing the uvula to right side along with a smooth overlying mucosa. FNAC confirmed the diagnosis pleomorphic adenoma. CT and MRI showed tumour measuring 3 × 4 cm in the left parapharyngeal space (Figure 5). A transcervical approach was used to gain entry into parapharyngeal space without any osteotomy of the mandible; the tumour was excised. Postoperative period was uneventful.

## 5. Discussion

Parapharyngeal space masses account for 0.5% of all head and neck tumours and the majority is histopathologically benign (76%) [1]. Neoplasms of salivary gland origin are located in the prestyloid parapharyngeal space (PPS) and account for 40–50% of parapharyngeal space lesions. Salivary neoplasms may arise from the deep lobe of the parotid gland, ectopic salivary rests, or minor salivary glands of the lateral pharyngeal wall [2].

Minor salivary gland tumours constitute 22% of all the salivary gland tumours and among them only 18% portion of them are histopathologically benign, the rest being malignant. Among the benign salivary gland tumours seen in the PPS the most common prestyloid lesion is pleomorphic adenoma, which represents 80–90% of salivary neoplasms in the parapharyngeal space [2].

According to a study the most common site of pleomorphic adenoma of the minor salivary gland is the palate followed by lip, buccal mucosa, floor of mouth, tongue, tonsil, pharynx, retromolar area, and nasal cavity [3]. Unlike most mixed tumors, those in the palate and lip frequently lack a capsule.

Pleomorphic adenoma arising from the epithelial rests of the salivary tissue in the lymph nodes of the parapharyngeal space is very rare. According to the literature, there are few cases reported up till now that have taken the origin from the minor salivary gland tissue of the PPS [4, 5]. 18% of the tumours arising in the minor salivary glands are benign and pleomorphic adenoma is the commonest of all [6].

In two of the cases reported in this study, the tumours originated from the minor salivary gland tissue of the PPS.

Pleomorphic adenoma may also invade PPS as an extension of the deep lobe of the parotid gland [7–9]. Parotid tissue can herniate through a weakness in the stylomandibular membrane and lie in the lateral pharyngeal wall. For this reason, tumors deep in the parotid gland can present as parapharyngeal masses. Almost 10–12% of pleomorphic adenomas of the parotid are thought to arise from the deep lobe of parotid and parapharyngeal extensions of the mass in the deep lobe may remain asymptomatic until reaching a very large size.

The diagnostic workup algorithm begins with haematologic and serologic tests. Followed by fine needle aspiration cytology (FNAC), CT, and/or MRI studies should be the part of the preoperative diagnosis to determine the extent of disease, local spread, and even the type of tumour. When the tumours arise from the deep lobe of the parotid, they can appear entirely extraparotid seen in the parapharyngeal space, without a fat plane between it and the parotid, and widen the stylomandibular tunnel. MRI has been shown to be superior to computed tomography in the investigation of parapharyngeal space tumours [10].

The treatment of these tumours is the most challenging part to the head and neck surgeon because of the difficult location of the tumour with very important structures nearby such as large vessels of the neck, sympathetic chain, lymph nodes, and lower cranial nerves [7]. It is also written that any of these vital structures can be involved with major or minor trauma resulting in undesired consequences [7]. However, the transoral approach is considered to be unsafe since it is correlated with many postoperative complications such as haemorrhage, fistulas, dehiscence, and nerve damage and, for these same reasons, the combined transoral-transcervical approach should also be avoided [11]; for benign prestyloid space tumours of <3 cm, a transoral approach can be adopted [12].

For resection of the benign neoplasm, there is also a new method suggested that endoscope-assited transoral approach provides less operative trauma [13].

When choosing the surgical treatment it is also advised to split the mandible in order to gain space to approach the tumour for a safer excision. However, in all of our cases, we performed the surgeries without splitting the mandible because the location of a benign salivary gland tumour in the parapharyngeal space, which is usually prestyloid, enables the excision of the tumour without compromising great vessels or the other vital structures that are situated posterior to the tumour, when approaching from inferior and anterior. Such an approach can easily be performed through a submandibular transcervical incision.

As for the deep lobe tumours, a transcervical submandibular approach is also mandatory and again no splitting on the mandible is needed.

In none of our cases mandibular splitting was performed. All the three cases were well encapsulated and they did not show any blushing when contrast studies were performed. One of the cases (Case 1) also underwent angiographic studies which showed no contrast blushing of the tumour as well. According to the preoperative FNACs, all of them were pleomorphic adenomas.

According to our study it can be concluded that, in selected cases of PPS benign tumours, the submandibular transcervical approach without an osteotomy can be as safe as an transcervical approach with split osteotomies of the mandible.

## References

[1] Carrau RL, Myers EN, Johnson JT (1990). Management of tumors arising in the parapharyngeal space. *The Laryngoscope*.

[2] Hughes KV, Olsen KD, McCaffrey TV (1995). Parapharyngeal space neoplasms. *Head and Neck*.

[3] Verghese BT, Sebastian P, Abraham EK, Mathews AA (2003). Case report: pleomorfic adenoma of minor salivary gland in the parapharengeal space. *World Journal of Surgical Oncology*.

[4] Bent JP, Dinges D, Whitehouse A (1992). Pathologic quiz case-Minor salivary gland pleomorphic adenoma of the parapharyngeal space. *Archives of Otolaryngology—Head and Neck Surgery*.

[5] Hakeem AH, Hazarika B, Pradhan SA, Kannan R (2009). Primary pleomorphic adenoma of minor salivary gland in the parapharyngeal space. *World Journal of Surgical Oncology*.

[6] Stanley RE (1991). Parapharyngeal space tumours. *Annals of the Academy of Medicine Singapore*.

[7] Sergi B, Limongelli A, Scarano E, Fetoni AR, Paludetti G (2008). Giant deep lobe parotid gland pleomorphic adenoma involving the parapharyngeal space. Report of three cases and review of the diagnostic and therapeutic approaches. *Acta Otorhinolaryngologica Italica*.

[8] Morita N, Miyata K, Sakamoto T, Wada T (1995). Pleomorphic adenoma in the parapharyngeal space: report of three cases. *Journal of Oral and Maxillofacial Surgery*.

[9] Work PW, Gates GA (1969). Tumours of parapharyngeal space. *Otolaryngologic Clinics of North America*.

[10] Lloyd GA, Phelps PD (1986). The demonstration of tumours of the parapharyngeal space by magnetic resonance imaging. *British Journal of Radiology*.

[11] Olsen KD (1994). Tumors and surgery of the parapharyngeal space. *Laryngoscope*.

[12] Bozza F, Vigili MG, Ruscito P, Marzetti A, Marzettt F (2009). Surgical management of parapharyngeal space tumours: results of 10-year follow-up. *Acta Otorhinolaryngologica Italica*.

[13] Iseri M, Ozturk M, Kara A, Ucar S, Aydin O, Keskin G (2014). Endoscope-assisted transoral approach to parapharyngeal space tumors. *Head & Neck*.

